# SNPs in lncRNA Regions and Breast Cancer Risk

**DOI:** 10.3389/fgene.2020.00550

**Published:** 2020-06-30

**Authors:** Maija Suvanto, Jonathan Beesley, Carl Blomqvist, Georgia Chenevix-Trench, Sofia Khan, Heli Nevanlinna

**Affiliations:** ^1^Department of Obstetrics and Gynecology, University of Helsinki and Helsinki University Hospital, Helsinki, Finland; ^2^Department of Genetics and Computational Biology, QIMR Berghofer Medical Research Institute, Brisbane, QL, Australia; ^3^Department of Oncology, University of Helsinki and Helsinki University Hospital, Helsinki, Finland; ^4^Turku Bioscience Centre, University of Turku and Åbo Akademi University, Turku, Finland

**Keywords:** breast cancer, lncRNA, ultraconserved region, GABPB1-AS1, breast cancer risk

## Abstract

Long non-coding RNAs (lncRNAs) play crucial roles in human physiology, and have been found to be associated with various cancers. Transcribed ultraconserved regions (T-UCRs) are a subgroup of lncRNAs conserved in several species, and are often located in cancer-related regions. Breast cancer is the most common cancer in women worldwide and the leading cause of female cancer deaths. We investigated the association of genetic variants in lncRNA and T-UCR regions with breast cancer risk to uncover candidate loci for further analysis. Our focus was on low-penetrance variants that can be discovered in a large dataset. We selected 565 regions of lncRNAs and T-UCRs that are expressed in breast or breast cancer tissue, or show expression correlation to major breast cancer associated genes. We studied the association of single nucleotide polymorphisms (SNPs) in these regions with breast cancer risk in the 122970 case samples and 105974 controls of the Breast Cancer Association Consortium’s genome-wide data, and also by *in silico* functional analyses using Integrated Expression Quantitative trait and *in silico* prediction of GWAS targets (INQUISIT) and expression quantitative trait loci (eQTL) analysis. The eQTL analysis was carried out using the METABRIC dataset and analyses from GTEx and ncRNA eQTL databases. We found putative breast cancer risk variants (*p* < 1 × 10^–5^) targeting the lncRNA *GABPB1-AS1* in INQUISIT and eQTL analysis. In addition, putative breast cancer risk associated SNPs (*p* < 1 × 10^–5^) in the region of two T-UCRs, uc.184 and uc.313, located in protein coding genes *CPEB4* and *TIAL1*, respectively, targeted these genes in INQUISIT and in eQTL analysis. Other non-coding regions containing SNPs with the defined p-value and highly significant false discovery rate (FDR) for breast cancer risk association were discovered that may warrant further studies. These results suggest candidate lncRNA loci for further research on breast cancer risk and the molecular mechanisms.

## Introduction

About 70–90% of the human genome is transcribed (Guttman et al., 2009; Mercer et al., 2009). The protein coding RNAs account for only a small fraction of all the transcripts, while non-coding RNAs (ncRNA) cover 95% (Dermitzakis et al., 2005; Kapranov et al., 2007; Mattick, 2009). These include long non-coding RNAs (lncRNAs), defined as ncRNAs with over 200 nucleotides. They participate in various biological processes, including differentiation, immune response and metabolism (Kretz et al., 2013; Hung et al., 2014; Wang et al., 2014) as well as in pathogenic processes, such as the development and progression of cancer (Gupta et al., 2010; Yang et al., 2013; Xing et al., 2014). Their expression exhibits cell type specificity and responds to various stimuli, suggesting a rigorous transcriptional regulation (Wang and Chang, 2011).

A curious subclass of lncRNAs are the ultraconserved regions (UCRs). These are stretches of DNA expanding over 200 nucleotides that are absolutely conserved between orthologous regions in human, mouse and rat (Bejerano et al., 2004). There exist 481 such regions spread across the human genome, and 93% of the UCRs are transcribed in at least one normal human tissue (Calin et al., 2007). However, the study of T-UCR expression is complicated: based on annotation compiled by Mestdagh et al. (2010), 38.7% of the 481 T-UCRs are intergenic and 57.4% of the 481 T-UCRs are located in protein coding genes (42.6% intronic, 4.2% exonic, 5% partly exonic, and 5.6% exon containing), and 3.9% of T-UCRs lack an explicit gene-related annotation, because of the host gene splice variants. For these intragenic T-UCRs, it is difficult to define if the expression signal/measurement comes from the T-UCR or from the host gene. Mestdagh et al. (2010) studied this question in neuroblastoma tissue and found 237 T-UCRs to be independently expressed while the expression of the remaining 244 T-UCRs was inseparable from the host gene expression, either because the T-UCR was expressed as a part of the host gene transcript, or because the T-UCR and host gene expressions correlate for some other reasons. Interestingly, many of the T-UCRs are located in cancer-related regions and fragile sites, and their expression is frequently altered in human cancer (Amos et al., 2017; Fabris and Calin, 2017; Terracciano et al., 2017).

Breast cancer is the most common cancer in women worldwide and the leading cause of female cancer deaths (Torre et al., 2015). Breast cancer risk has a strong hereditary aspect, especially genes encoding tumor suppressors, which play a role in DNA damage response and repair pathways, are mutated in hereditary breast cancer (Goldgar et al., 1994; Lichtenstein et al., 2000; Collaborative Group on Hormonal Factors in Breast Cancer, 2001; Nielsen et al., 2016). *BRCA1* and *BRCA2* genes carry pathogenic variants of high-penetrance that cover approximately 20% of the familial relative risk (Mavaddat et al., 2015). Other variants, the majority of them with moderate or low penetrance, have been found to cover little over 20%, putting the altogether familial relative risk coverage to approximately 44% (Michailidou et al., 2017). Up to the present, nearly 200 low-penetrance susceptibility loci have been identified. While high- and moderate-penetrance variants are often in protein coding regions, low-penetrance variants are typically located in non-coding regions (Ghoussaini et al., 2013; Michailidou et al., 2017; Milne et al., 2017).

Recently, several studies have shown a link between genetic variants in lncRNA genes and breast cancer risk. Cui et al. (2018) found a SNP 2 kb upstream of H19 transcription start site that was associated with breast cancer risk in estrogen receptor (ER)-positive patients in the Chinese population. Wu et al. (2018) studied risk associations among 22977 cases and 105974 controls of European ancestry and found several novel risk-loci that harbored lncRNA genes. Three of these lncRNAs, and four altogether (*ANRIL*, *H19*, *PVT1*, and *IGF2-AS*), were reported to have disease association based on SNP-association either with breast cancer or prostate cancer risk or survival (Eeles et al., 2009; Turnbull et al., 2010; Meyer et al., 2011; Riaz et al., 2012). In addition, several lncRNAs have been found to be differentially expressed in various breast cancer subtypes (Mathias et al., 2019). While the precise functionality of lncRNAs in breast cancer remains to be elucidated, they play a role in the regulation of intracellular and intercellular signaling (Klinge, 2018).

The Breast Cancer Association Consortium (BCAC) is an international multidisciplinary consortium with a focus on inherited risk of breast cancer^1^. Their aim is to combine data from many studies to identify genes related to breast cancer risk and, with the world’s largest collection of breast cancer case samples and controls, provide a powerful assessment of risk associated with the studied genes. BCAC has the largest genomic breast cancer dataset worldwide. Several papers describe in detail BCAC and genotyping projects using the BCAC dataset (Michailidou et al., 2013, 2015, 2017).

In this study, we look into the breast cancer risk association of SNPs on lncRNAs expressed in mammary tissue or associated with known breast cancer risk genes, as well as SNPs located at the T-UCRs. We carried this out by analyzing the Breast Cancer Association Consortium’s (BCAC) GWAS, OncoArray, and iCOGs SNP array summary statistics to find SNPs in or near lncRNAs or T-UCRs that associate with breast cancer risk. The loci with GWAS-significant results have been published recently (Michailidou et al., 2017; Milne et al., 2017), and in this study we concentrate on the lncRNA and T-UCR related SNPs with *p* < 10^–5^ to uncover other candidate lncRNA loci for further analysis. The functionality of the SNPs of interest was studied with integrated expression quantitative trait and *in silico* prediction of GWAS targets (INQUISIT; Michailidou et al., 2017) and eQTL analysis. We found putative breast cancer risk variants associated with the expression of lncRNA GA-binding protein transcription factor beta subunit 1 antisense RNA 1 (*GABPB1-AS1*), cytoplasmic polyadenylation element binging protein 4 (*CPEB4*) associated with uc.184, and TIA 1 cytotoxic granule associated RNA binding protein like 1 (*TIAL1*) associated with uc.313.

## Materials and Methods

**FIGURE 1:**
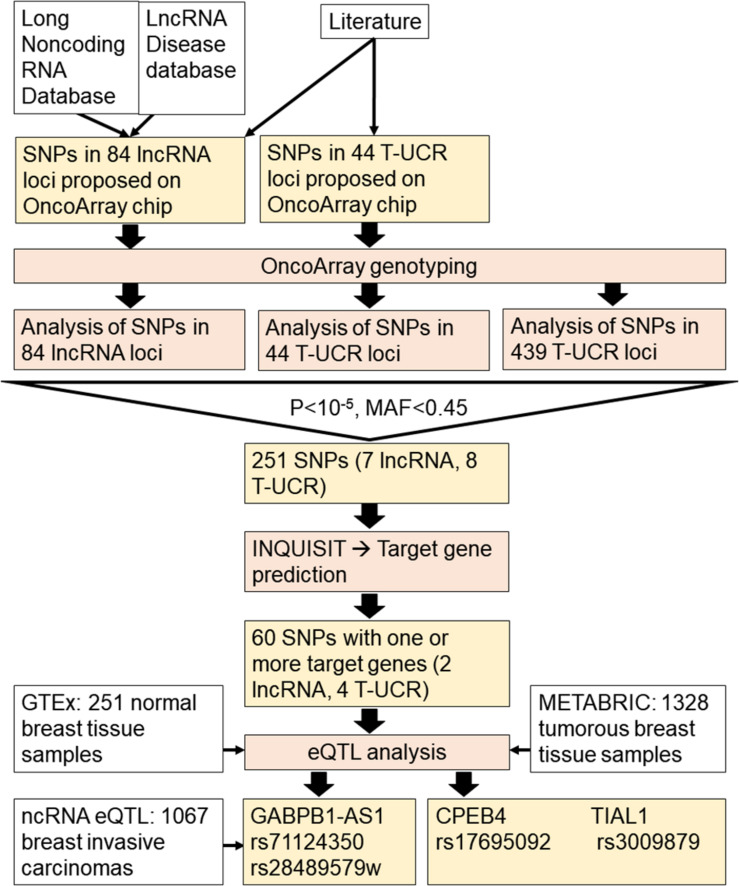
Workflow chart of the study.

The work flow of the study is presented in Figure 1.

### Study Population

The analyses were based on summary results of the Breast Cancer Association Consortium (BCAC). The collaborative dataset of the BCAC contained 122970 female breast cancer case samples and 105974 controls of European ethnicity. Of these, 61282 cases and 45494 controls were genotyped using OncoArray (Amos et al., 2017), and 46785 cases and 42892 controls using iCOGs (Michailidou et al., 2013), while 14910 cases and 17588 controls came from 11 other breast cancer GWAS experiments (Michailidou et al., 2015). All participating studies were approved by their appropriate institutional ethics review board and all subjects provided informed consent. All research was performed in accordance with the relevant guidelines and regulations.

### Selection of lncRNA Regions for the Study

We selected 565 regions of lncRNAs and T-UCRs. Following a comprehensive search for relevant lncRNAs we selected altogether 84 lncRNA regions with reported polymorphisms based on multiple criteria including tissue specific expression, positive expression correlation with high and moderate penetrance genes, and known disease associations (Supplementary Table S1). 46 lncRNAs had expression above five tags per million (Gibb et al., 2011) in breast tumor tissue. Ten of these, and 25 other lncRNAs, showed positive expression correlation with high and moderate penetrance genes (ten with *BRCA1*, three with *BRCA2*, two with *ATM*, one with *CDH1*, three with *CHEK2*, two with *PALB2*, thirteen with *RAD51C*, and one with *TP53*). Several lncRNAs showed positive correlation with multiple of these genes, but here only the strongest correlations are listed (Supplementary Table S1). For the correlation analysis, we used expression data from GENCODE. The expression data as normalized RPKM (reads per kilobase per million mapped reads) values was retrieved from GENCODE database v7 (Derrien et al., 2012). Twenty-two lncRNAs had a reported disease association defined either by higher expression in a tumor tissue compared to a normal tissue or by chromosomal aberrations in lncRNA regions in samples from breast, ovarian or prostate cancer (data retrieved from Long Non-coding RNA Database (Amaral et al., 2011), LncRNADisease database (Chen et al., 2013) and literature in March 2013) (Supplementary Table S2). Three of these lncRNAs, and four altogether (*ANRIL, H19, PVT1*, and *IGF2-AS*) were reported to have disease association based on SNP association either with breast cancer or prostate cancer risk or survival (Eeles et al., 2009; Turnbull et al., 2010; Meyer et al., 2011; Riaz et al., 2012). For these 84 lncRNAs we included SNPs located in exons and 50 kb flanking regions, 5′UTRs, and 150 nucleotides upstream from a transcription starting site. The SNPs in the 84 lncRNA regions were genotyped on the OncoArray genotyping chip (Amos et al., 2017). In addition, we selected 44 T-UCR regions that were either highly expressed in normal breast tissue and/or had a known enhancer activity and/or were located at cancer-associated genomic regions (Calin et al., 2007; Scaruffi, 2011) (Supplementary Table S3). SNPs in these T-UCR loci, including 50 bp extended region on both sides, with 1000 genomes European MAF ≥ 0.0013 were selected for genotyping on the OncoArray.

Here, we have included in the analysis all the genotyped SNPs in the 84 lncRNA regions and 44 T-UCR regions, and extended our study to also include the remaining T-UCR regions resulting in an extensive explorative study of all the 481 T-UCR regions in the genome (Bejerano et al., 2004). While Bejerano et al. (2004) reported no evidence that 256 of these 481 ultraconserved regions were transcribed, Calin et al. (2007) found that 93% of these regions were transcribed in at least one normal human tissue. Thus we decided to include all ultraconserved regions in this study alongside the other lncRNAs, as well as to refer to them as T-UCRs.

The regions of interest that were used to gather SNPs from the BCAC results database were defined as the above mentioned 565 lncRNA or T-UCR of interest, and 50 kb flanking it in both directions.

### Genotyping

OncoArray contains approximately 533000 markers, while iCOGS holds 211000 (18, 19). Their genotyping and the genotyping of the eleven GWAS in the BCAC has been previously described in detail (Michailidou et al., 2013, 2015, 2017). All samples were imputed using the version 3 (October 2014) release of the 1000 Genomes Project dataset as the reference panel. For iCOGS, OncoArray, and nine of the eleven GWAS, the imputation was carried out with a 2-stage approach using SHAPEIT2 for phasing and IMPUTE v2 for imputation; the two remaining GWAS were imputed separately using MaCH and Minimac (Howie et al., 2009, 2012; Li et al., 2010; O’Connell et al., 2014). The details of the imputation process have been described previously (Michailidou et al., 2017). Summary statistics used in the study were obtained through BCAC. In this study, we looked at associations in 565 specific regions, and used a *p*-value of *p* < 10^–5^ as the limit of interrogation.

### Target Gene Prediction

The functionality of the putative breast cancer risk variants was assessed by annotating each variant with publicly available genomic data from breast cells and by using a heuristic scoring system (Integrated Expression Quantitative trait and *in silico* prediction of GWAS targets, INQUISIT) that combines genomic data from multiple sources, including chromatin interactions, computational enhancer–promoter correlations, transcription factor binding chromatin immunoprecipitation followed by sequencing, gene expression and topologically associated domain boundaries, and which is described in detail by Michailidou et al. (2017). For this study, the target gene predictions were made from annotation in MCF7 and HMEC cells, and the prediction methods were chromatin interaction analysis by paired-end tag sequencing (ChIA-PET), integrated methods for predicting enhancer targets (IM-PET) and analysis of super-enhancers as defined by Hnisz et al. (2013).

### Expression Quantitative Trait Loci (eQTL) Analysis

The Genotype-Tissue Expression (GTEx) project’s breast tissue eQTL results (version 7) were used to detect SNP associations with gene expression. The dataset included 251 normal breast tissue samples. The data used for the analyses in this study were downloaded from the GTEx Portal^2^ on February 13th, 2018 (version 7).

In addition, an eQTL analysis of the Molecular Taxonomy of Breast Cancer International Consortium (METABRIC, Curtis et al., 2012) dataset was carried out. The raw genotype data (Affymetrix SNP 6.0 platform) and normalized mRNA expression data (Illumina HT-12 v3 platform) extracted from matched DNA and RNA specimens of tumorous breast tissue were downloaded from the European Genome-phenome Archive^3^. The genotype data was processed with Affymetrix Genotyping Console Software following the practices of the Affymetrix SNP 6.0 analysis workflow. The workflow including a quality control step has been previously described (Jamshidi et al., 2015; Khan et al., 2015). After the quality control, the analysis contained 1328 samples with both genotype and expression data. The analysis was carried out using R-package Matrix eQTL with linear regression model (Shabalin, 2012).

The recently published database ncRNA eQTL was queried to validate *GABPB1-AS1* eQTL results^4^ (Li et al., 2019).

### Statistical Analysis

The BCAC summary results included a meta-analysis of OncoArray, iCOGS and 11 GWAS analyses, as well as effect size and standard error and *p*-value for these analyses for all variants. The meta-analysis has been described in detail in Michailidou et al. (2017) and the summary results are available at^5^. An FDR cut-off of 0.5 was used to evaluate the importance of the findings. FDR was calculated with the Benjamini–Hochberg procedure for all SNPs in the regions of interest using the R 3.5.2 environment (R Core Team, 2013)^6^.

Statistical analysis of INQUISIT is described in detail in Michailidou et al. (2017).

For the eQTL analysis results, a cut-off of nominal *p* = 0.05 was used. The eQTL data available at the GTEx Portal included *p*-values, normalized size effect (NES) and standard error to NES. The R-package Matrix eQTL used to carry out METABRIC eQTL analysis also provided FDR *p*-values. Those were viewed as additional information for the discussion. ncRNA eQTL statistical information included beta, r, and *p*-values.

### Online Bioinformatics Tools

Linkage disequilibrium between SNPs was checked using Broad Institutes SNP annotation and proxy search (SNAP v2.2; Johnson et al., 2008) and LDlink (v3.7; Machiela and Chanock, 2015). Promoter/enhancer loci were browsed using GeneHancer in GeneCards (^7^, version 4.7, accessed 4.5.2018, Fishilevich et al., 2017). Gene and SNP positions were checked using the UCSC Genome Browser (^8^, Kent et al., 2002) and Ensembl genome browser 92 (^9^, Zerbino et al., 2018).

## Results

In this study, we looked into the breast cancer risk association of SNPs in the regions of breast cancer-relevant lncRNAs and of T-UCRs around the genome in a large cohort of European breast cancer patients. We selected altogether 565 lncRNA regions that included 84 lncRNAs with reported polymorphisms based on multiple criteria, including tissue specific expression, co-expression with high and moderate penetrance genes, and known disease associations, and 481 ultraconserved regions. 153 ultraconserved regions including the 44 T-UCRs selected for the OncoArray were either highly expressed in normal breast tissue and/or had a known enhancer activity and/or were located at cancer-associated genomic regions while no such information was available for the rest of the ultraconserved regions. The regions of interest were defined as the transcribed lncRNA/T-UCR and 50 kb up- and downstream genomic sequence. For the sake of brevity and clarity, we numbered the lncRNA regions and refer to those numbers in this article instead of the subject lncRNA of the region. The nomenclature for the ultraconserved regions came from the article by Bejerano et al. (2004). All regions, genes and the rationales for selecting them for this study can be found in Supplementary Table S1 and Supplementary Table S2. Positional annotations follow Human GRCh37/Hg19.

### SNPs in Seven lncRNAs and Eight T-UCRs Associated With Breast Cancer Risk

We used BCAC summary statistics on risk results from meta-analysis of OncoArray, iCOGS and 11 separate genome-wide association studies (GWAS). The regions of interest included 5401 genotyped and 349112 imputed SNPs. Results with genome-wide significance level (*p* < 5 × 10^–8^) for five of the lncRNA regions and 18 of the T-UCR regions have previously been published by the BCAC and are listed in Supplementary Table S4. These regions are undergoing further fine mapping studies by the BCAC. Here, a *p*-value of <10^–5^ and MAF <0.45 was used as the limit of interrogation, resulting in seven lncRNA regions and eight T-UCRs containing three genotyped and 248 imputed SNPs not previously reported by the BCAC (Tables 1, 2). FDR was calculated for all the SNPs in the regions of interest to evaluate the importance of the findings (Supplementary Table S5). None of the SNPs in the T-UCR regions were directly in the T-UCRs themselves, but in the regions flanking them. This is expected due to the nature of ultraconservation, but makes it difficult to analyze the relationship between the SNP and the T-UCR.

**TABLE 1:** All lncRNA regions where SNPs with *p* < 10^–5^ were found.

**Regions**	**Position**	**Subject of the region**	**Subject associated gene**	**Subject position**	**Subject rationale**	**FANTOM-CAT gene category based on DHS support (DHS support in paranthesis)**	**FANTOM-CAT gene class based on coding potential and genomic context**
lncRNA-45	chr3:177484653-177667012	ENSG000 00231574	RP11-91K9.1	chr3:177534653-177617012	LncRNA expression positively correlates with a BC high/moderate penetrance gene expression	e-lncRNA (enhancer)	lncRNA, intergenic
lncRNA-49	Chr6:33167311-33272766	ENSG00 000232940		chr4:33217311-33222766	LncRNA expression positively correlates with a BC high/moderate penetrance gene expression	others (dyadic)	lncRNA, divergent
lncRNA-103	chr8:17689552-17790170	ENSG00 000253215		chr8:17739552-17740170	LncRNA expression positively correlates with a BC high/moderate penetrance gene expression	others (no)	ncRNA, divergent
lncRNA-26	chr10:38403576-38551285	ENSG00 000224761	RP11-508N22.8	chr10:38453576-38501285	LncRNA higly expressed in breast tumor tissue	not in FANTOM-CAT db	not in FANTOM-CAT db
lncRNA-82	chr11:18571334-18681802	ENSG000 00247595	RP11-504G3.1	chr11:18621334-18631802	LncRNA moderately expressed in breast tumor tissue and its expression positively correlates with RAD50 expression	p-lncRNA (promoter)	lncRNA, divergent
lncRNA-17	chr14:101195747-101377368	ENSG00 000214548	MEG3	chr14:101245747-101327368	LncRNA higly expressed in breast tumor tissue	p-lncRNA (promoter)	lncRNA, intergenic
lncRNA-75	chr15:50597156-50714399	ENSG0000 0244879	GABPB1-AS1	chr15:50646371-50650503	LncRNA expression positively correlates with BRCA1 expression	p-lncRNA (promoter)	lncRNA, divergent

**TABLE 2:** All ultraconserved regions where SNPs with *p* < 10^–5^ were found.

**Region**	**Position**	**T-UCR position**	**Genomic annotation**	**Host gene**	**Host gene name**	**Target rationale**
uc.61	chr2:60637573-60737898	chr2:60687573-60687898	part_exonic/exonic	NM_018014/NM_ 138559/NM_022893	BCL11A	T-UCR
uc.147	chr4:151186383-151286690	chr4:151236383-151236690	intronic	NM_006726	LRBA	T-UCR
uc.148/149	chr4:151443952-151544396	chr4:151493952-151494191, chr4:151494193-151494396	intronic	NM_006726	LRBA	T-UCR
uc.184	chr5:173335292-173435521	chr5:173385292-173385521	3′UTR	NM_030627/CPEB4	CPEB4	T-UCR
uc.201	chr6:100001984-100102223	chr6:100051984-100052223	intergenic	na	na	T-UCR
uc.250	chr9:13889910-13990118	chr9:13939910-13940118	intronic	XM_001724969	RP11-284P20.1	T-UCR
uc.313	chr10:121290174-121390404	chr10:121340174-121340404	intronic	XM_001718650/NM_ 001033925/NM_003252	TIAL1	T-UCR
uc.386	chr15:37472009-37572211	chr15:37522009-37522211	intergenic	na	na	T-UCR Located at Cancer-associated genomic regions

In addition to the results from the meta-analysis of breast cancer overall, we interrogated the meta-analysis results from ER-negative and ER-positive patient subgroups separately (Table 3). Fourteen SNPs (all imputed) were shared between the overall and ER-negative analyses, all located in the uc.147 region, and 5 SNPs had *p* < 10^–5^ in the ER-negative analysis only (all imputed). Nine SNPs were shared between the overall and ER-positive analyses (all imputed) and no SNP gave a *p*-value under the threshold in ER-positive analysis only. None of the SNPs were shared by all three subgroup analyses.

**TABLE 3:** Distribution of SNPs with *p* < 10^–5^ to regions. BC, breast cancer. MA, meta-analysis.

**Regions**	**Novel region**	**SNPs**	**Genotyped/imputed**	**SNPS in MA of overall BC**	**SNPs in MA of ER-**	**SNPs in MA of ER+**
lncRNA-2	No	125	23/102	125	0	110
lncRNA-17	Yes	2	0/2	2	0	0
lncRNA-26	Yes	20	0/20	20	0	0
lncRNA-43	No	129	9/120	114	0	107
lncRNA-45	Yes	1	0/1	1	0	0
lncRNA-49	Yes	1	0/1	0	1	0
lncRNA-69	No	234	122/112	189	70	116
lncRNA-75	Yes	20	3/17	20	0	0
lncRNA-82	Yes	1	0/1	1	0	1
lncRNA-92	No	34	15/19	32	0	32
lncRNA-98	No	16	11/5	13	0	16
lncRNA-103	Yes	9	0/9	9	0	0
uc.1	No	180	0/180	180	179	52
uc.2	No	2	0/2	2	1	0
uc.2/3	No	2	0/2	2	0	0
uc.2/3/4	No	9	0/9	7	2	0
uc.22/23	No	1	0/1	1	0	0
uc.23/24	No	1	0/1	1	0	0
uc.24	No	2	0/2	2	0	0
uc.24/25	No	2	0/2	2	0	0
uc.25	No	7	0/7	7	0	0
uc.29	No	11	0/11	1	0	11
uc.61	Yes	16	0/16	16	0	0
uc.98/99	No	11	0/11	8	0	11
uc.147	Yes	32	0/32	29	17	0
uc.148/149	Yes	1	0/1	0	1	0
uc.152	No	2	0/2	1	0	2
uc.162	No	33	5/28	33	15	0
uc.168	No	15	2/13	15	0	0
uc.175	No	85	0/85	85	23	79
uc.184	Yes	57	0/57	57	0	0
uc.201	Yes	1	0/1	1	0	0
uc.245	No	57	0/57	52	0	57
uc.250	Yes	43	0/43	43	0	0
uc.313	Yes	46	0/46	46	0	8
uc.386	Yes	1	0/1	1	0	0
uc.401	No	60	0/60	5	15	55

### Integrated Expression Quantitative Trait and *in silico* Prediction of GWAS Targets (INQUISIT) Predicts Target Genes for 60 SNPs in Two lncRNAs and Four T-UCRs

A heuristic scoring system, INQUISIT (Michailidou et al., 2017), was used to calculate the potential target genes for the 251 SNPs that were associated with breast cancer risk in BCAC analysis (Supplementary Table S6).

For 60 of the 251 SNPs, INQUISIT predicted one or more target genes (Supplementary Table S7). There were 12 genes predicted as targets altogether and each gene had 1-17 SNPs predicting it. The SNPs resided on two lncRNAs regions and on four T-UCRs; the number of SNPs per region ranged from 1-22. The predominant method of prediction was chromatin interaction analysis by paired-end tag sequencing (ChIA-PET) in MCF7. All these SNPs had association (*p* < 10^–5^) with overall breast cancer, with FDR <0.005. Four SNPs in uc.313 were also associated with ER-positive breast cancer. It is to be noted that MCF7 is a breast cancer cell line which may cause alteration in its cellular processes and may affect these results.

Overall, the INQUISIT-predicted target genes of the SNPs were not the lncRNA or T-UCR of the SNPs region, but mostly protein-coding genes. The only exception to this was *GABPB1-AS1*, the subject of the lncRNA-75 region and targeted by three lncRNA-75 SNPs in INQUISIT predictions. Three T-UCRs were located within the gene that the SNPs in their regions targeted by INQUISIT: uc.147 in the intron of *LRBA*, uc.184 in the 3′UTR of *CPEB4* and uc.313 in the intron of *TIAL1*.

We searched for genes that, in addition to being INQUISIT target genes, show eQTL associations as well (see below). There were three such genes in our data: *GABPB1-AS1* (GTEx eQTL analysis), *CPEB4* and *TIAL1* (METABRIC eQTL analysis) (Table 4). Only in a few cases, the SNP targeting a gene in INQUISIT predictions was the same SNP that associates to the gene in eQTL (Table 5). However, the majority of these SNPs are linked.

**TABLE 4:** Genes that are both INQUISIT predicted target genes and have eQTL associations.

**Gene**	**Region**	**SNPs in INQUISIT**	**SNPs in METABRIC**	**SNPS in GTEx**
GABPB1-AS1	lncRNA-75	3		18
CPEB4	uc.184	17	2	
TIAL1	uc.313	10	3	

**TABLE 5:** SNPs that both have INQUISIT predicted target genes and have eQTL associations.

**Variant**	**Gene**	**Region**	**MAF**	**OR (95%CI) BCAC**	**p BCAC**	**p eQTL**	**eQTL**
rs71124350	GABPB1-AS1	lncRNA-75	0.3354	0.97 (0.957–0.983)	0.00000784	2.41918E-07	GTEx
rs28489579	GABPB1-AS1	lncRNA-75	0.3444	0.97 (0.957–0.983)	0.000005277	1.56507E-13	GTEx
rs17695092	CPEB4	uc.184	0.3149	0.969 (0.956–0.982)	0.000002644	7.33215E-61	METABRIC
rs3009879	TIAL1	uc.313	0.4089	1.03 (1.017–1.043)	0.000003064	0.001347099	METABRIC

Three SNPs targeted *GABPB1-AS1* of lncRNA-75 region (rs1806845, rs71124350, and rs28489579) (Table 6). All three clustered together approximately 31 kb downstream of the lncRNAs. These SNPs also have additional predicted targets, rs1806845 and rs71124350 target also *SLC27A2* and *GABPB1* and rs28489579 targets *GABPB1* as well. However, none of these other predicted targets show association with *p* < 0.05 in the eQTL analyses.

**TABLE 6:** Variants with GABPB1-AS1 as INQUISIT predicted target gene and/or GTEx eQTL association.

**Variant**	**Chr**	**Position**	**Position in relation to GABPB1-AS1**	**Alleles**	**Breast cancer risk (BCAC) (meta-analysis, all samples)**				**INQUISIT?**	**GTEx**			
					**OR (95 %CI)**	**SE**	***p***	**FDR**			**NES**	**NES SE**	***p***
rs606118	15	50655171	upstream	C/T	0.971 (0.9588–0.9840)	0.0066	0.000009757	0.00228261707542373	No	Yes	0.53	0.06	5.2 × 10−14
rs11634585	15	50656449	upstream	G/A	0.971 (0.9591–0.9839)	0.0065	0.000009465	0.00223705437071918	No	Yes	0.51	0.06	4.9 × 10−14
rs1056682	15	50660201	upstream	A/G	0.971 (0.9588–0.9840)	0.0066	0.000009377	0.0022257837394669	No	Yes	−0.53	0.06	5.7 × 10−14
rs17431150	15	50663582	upstream	G/A	0.971 (0.9587–0.9839)	0.0066	0.000008556	0.00208100765814978	No	Yes	−0.53	0.06	4.5 × 10−14
rs17431171	15	50663621	upstream	G/A	0.971 (0.9587–0.9839)	0.0066	0.00000855	0.00208100765814978	No	Yes	−0.53	0.06	5.2 × 10−14
rs34565064	15	50663903	upstream	G/A	0.971 (0.9591–0.9839)	0.0065	0.000009597	0.00226098220392492	No	Yes	−0.51	0.06	4.9 × 10−14
rs720599	15	50664515	upstream	C/A	0.971 (0.9586–0.9838)	0.0066	0.000007992	0.00196285368683274	No	Yes	−0.53	0.06	5.2 × 10−14
rs12905736	15	50667009	upstream	G/A	0.968 (0.9548–0.9822)	0.0072	0.000008952	0.00215079396344648	No	Yes	−0.54	0.07	4.2 × 10−12
rs55948407	15	50668901	upstream	G/A	0.970 (0.9575–0.9826)	0.0066	0.000004237	0.0011675312003988	No	Yes	−0.51	0.07	4.2 × 10−13
rs55941574	15	50668966	upstream	C/T	0.970 (0.9569–0.9824)	0.0067	0.000004257	0.00117049267828685	No	Yes	−0.5	0.07	1.5 × 10−11
rs35628775	15	50671563	upstream	T/C	0.970 (0.9579–0.9830)	0.0066	0.00000573	0.00149755046168401	No	Yes	−0.52	0.07	1.6 × 10−13
rs35541701	15	50673001	upstream	G/A	0.970 (0.9569–0.9824)	0.0067	0.000004296	0.00117886766600398	No	Yes	−0.51	0.07	8.2 × 10−13
rs34174311	15	50673281	upstream	T/C	0.971 (0.9582–0.9833)	0.0066	0.000006915	0.00174424701186131	No	Yes	−0.52	0.07	1.6 × 10−13
rs4775880	15	50675818	upstream	A/G	0.970 (0.9576–0.9831)	0.0067	0.000005656	0.00148561217126546	No	Yes	0.46	0.07	5.9 × 10−11
rs28817272	15	50677213	upstream	G/A	0.970 (0.9575–0.9830)	0.0067	0.000005417	0.00143237621551724	No	Yes	−0.46	0.07	2.6 × 10−11
rs1806845	15	50681906	upstream	G/T	0.971 (0.9580–0.9835)	0.0067	0.000009599	0.00226098220392492	Yes	No			
rs71124350	15	50682294	upstream	T/TA	0.970 (0.9572–0.9831)	0.0068	0.00000784	0.00193412589812332	Yes	Yes	−0.43	0.08	2.4 × 10−7
rs28489579	15	50686778	upstream	G/C	0.970 (0.9574–0.9829)	0.0067	0.000005277	0.00140477607425265	Yes	Yes	−0.52	0.07	1.6 × 10−13
rs4774565	15	50694306	upstream	A/G	0.968 (0.9546–0.9808)	0.0069	0.000002194	0.000674464429844098	No	Yes	−0.43	0.06	5.9 × 10−11

*NES, normalized effect size.*

*CPEB4* was the predicted target of seventeen SNPs in the uc.184 region (Table 7). The majority of the SNPs as well as uc.184 itself are located in the 3′UTR of the *CPEB4.* None of the SNPS overlap with uc.184. Two SNPs targeting *CPEB4* also had other predicted targets, *C5orf47* (rs17695092) and *NGS2* (rs55946741).

**TABLE 7:** Variants with CPEB4 as INQUISIT predicted target gene and/or METABRIC eQTL association.

**Variant**	**Chr**	**Position**	**Position in relation to CPEB4**	**Linkage**	**Alleles**	**Breast cancer risk (BCAC) (meta-analysis, all samples)**				**INQUISIT?**	**METABRIC**		
				**r^2^**		**OR (95 %CI)**	**SE**	**p**	**FDR**			**Beta**	***p* (FDR corrected)**
rs17695092	5	173337603	intron 2	1	T/G	0,969 (0,9563–0,9818)	0.0067	2.644E-06	0.0007848	Yes	Yes	−0.31702	3.75723E-55
3′UTR start	5	173385302											
rs1564823	5	173383194	3′UTR	1	C/A	0,968 (0,9553–0,9807)	0.0067	1.062E-06	0.0003778	No	Yes	0.32461	3.75057E-58
rs7736263	5	173339222	3′UTR	0.986	G/T	0,968 (0,9556–0,981)	0.0067	1.35E-06	0.0004578	Yes	No		
rs112299234	5	173339531	3′UTR	1	T/C	0,968 (0,9553–0,9807)	0.0067	1.038E-06	0.0003717	Yes	No		
rs72812804	5	173340496	3′UTR	1	T/G	0,968 (0,9557–0,9811)	0.0067	1.428E-06	0.0004738	Yes	No		
rs72812805	5	173344153	3′UTR	1	T/C	0,968 (0,9557–0,9811)	0.0067	1.436E-06	0.000474	Yes	No		
rs55946741	5	173345023	3′UTR	1	A/G	0,969 (0,9559–0,9813)	0.0067	1.667E-06	0.0005357	Yes	No		
rs10516107	5	173348156	3′UTR	1	G/A	0,968 (0,9557–0,9811)	0.0067	1.465E-06	0.0004795	Yes	No		
rs72812811	5	173350990	3′UTR	1	G/A	0,968 (0,9557–0,9811)	0.0067	1.465E-06	0.0004795	Yes	No		
rs17695555	5	173351081	3′UTR	1	C/T	0,968 (0,9557–0,9811)	0.0067	1.466E-06	0.0004795	Yes	No		
rs17763083	5	173351488	3′UTR	1	C/T	0,968 (0,9557–0,9811)	0.0067	1.463E-06	0.0004795	Yes	No		
rs747472	5	173353514	3′UTR	0.273	T/C	0,968 (0,9557–0,9811)	0.0067	1.419E-06	0.0004726	Yes	No		
rs17076726	5	173363889	3′UTR	0.995	C/T	1,03 (1,0172–1,0426)	0.0063	3.108E-06	0.000891	Yes	No		
rs56245789	5	173365310	3′UTR	1	T/C	0,968 (0,9553–0,9807)	0.0067	1.057E-06	0.0003765	Yes	No		
rs56196025	5	173365448	3′UTR	1	C/T	0,968 (0,9557–0,9811)	0.0067	1.431E-06	0.0004738	Yes	No		
uc.187	5	173385302-173385521	3′UTR										
3′UTR stop	5	173387994											
rs6890591	5	173392315	Upstream	0.672	T/A	0,972 (0,9598–0,9842)	0.0064	9.893E-06	0.0023027	Yes	No		
rs6869166	5	173392403	Upstream	0.68	A/G	0,972 (0,9597–0,9841)	0.0064	9.193E-06	0.0021953	Yes	No		
rs67623241	5	173392638	Upstream	0.674	C/G	0,971 (0,9593–0,9837)	0.0064	6.857E-06	0.0017337	Yes	No		

*Linkage of SNPs is to rs17695092 as the SNP with INQUISIt and eQTL results. BC, breast cancer.*

*TIAL1* was targeted by ten SNPs (Table 8). The target T-UCR, uc.313, overlaps none of the SNPs. Three SNPs also target *RGS10* (rs2917941, rs3009877, rs75611822).

**TABLE 8:** Variants with TIAL1 as INQUISIT predicted target gene and/or METABRIC eQTL association.

**Variant**	**Chr**	**Position**	**Position in relation to TIAL1**	**Linkage**	**Alleles**	**Breast cancer risk (BCAC) (meta-analysis, all samples)**				**INQUISIT?**	**METABRIC**		
				**r2**		**OR (95%CI)**	**SE**	**p**	**FDR**			**Beta**	**p**
rs10886511	10	121307823	downstream	0.493	G/A	1,033 (1,0186–1,0485)	0.0074	7.84E-06	1.93E-03	Yes	No		
rs10787979	10	121307837	downstram	0.365	G/A	1,037 (1,0221–1,0522)	0.0074	7.71E-07	2.91E-04	Yes	No		
rs4752331	10	121323976	downstram	0.702	A/G	1,033 (1,0194–1,0465)	0.0067	1.22E-06	4.24E-04	No	Yes	−0.04	0.007772
rs2917941	10	121328421	downstram	0.716	A/T	1,034 (1,0204–1,0471)	0.0066	5.46E-07	2.13E-04	Yes	No		
rs3009877	10	121328495	downstram	0.716	G/A	1,034 (1,0205–1,0472)	0.0066	5.18E-07	2.03E-04	Yes	No		
rs75611822	10	121329179	downstram	0.407	ACT/A	1,035 (1,0196–1,05)	0.0075	5.06E-06	1.36E-03	Yes	No		
rs10712346	10	121332690	downstram	0.996	AC/A	1,03 (1,0174–1,0433)	0.0064	3.91E-06	1.09E-03	Yes	No		
rs72040146	10	121332870	downstram	0.996	TAA/T	1,031 (1,0177–1,0436)	0.0064	3.03E-06	8.73E-04	Yes	No		
TIAL1 stop	10	121332978											
rs3009879	10	121338507	intron 6/7	1	C/T	1,03 (1,0174–1,0433)	0.0064	3.06E-06	8.80E-04	Yes	Yes	0.04	0.001347
uc.313	10	121340174–121340404	intron 5/6										
rs3816145	10	121347329	intron 2/3	0.409	T/C	1,033 (1,0184–1,0483)	0.0074	9.83E-06	2.29E-03	Yes	No		
rs146020828	10	121347839	intron 1/2	0.378	TTTTC/T	1,036 (1,0213–1,0514)	0.0074	1.45E-06	4.77E-04	Yes	No		
TIAL1 start	10	121356541											
rs12569630	10	121362660	upstream	0.955	A/G	1,031 (1,018–1,0439)	0.0064	1.69E-06	5.42E-04	No	Yes	−0.04	0.002971

*BC, breast cancer. Linkage of SNPs is to rs3009879 as the SNP with INQUISIT and eQTL results.*

### Two SNPs Targeting GABPB1-AS1 in INQUISIT Also Associate With It in eQTL Analysis of GTEx Dataset

GTEx eQTL association in normal mammary tissue with the limit of *p* < 0.05 was found for 171 of the 251 SNPs in this study (Supplementary Table S8). All in all, the SNPs had 318 associations with 22 genes. The SNPs were found on seven lncRNA and five T-UCR regions (Supplementary Table S9). Each SNP showed association to 1–4 genes and each gene to 1–48 SNPs. Only one gene, *GABPB1-AS1*, which was targeted in INQUISIT predictions, also had SNP association in GTEx analysis. *GABPB1-AS1* was also the only SNP associated gene that was also the target of a region of interest, lncRNA-75.

*GABPB1-AS1* expression associated with 18 SNPs in lncRNA-75 with p-values ranging from 2.45 × 10^–7^ to 4.47 × 10^–14^, and FDR corrected *p*-values all below 0.05 (Table 6). Curiously, all 36 associations in GTEx data with FDR corrected *p* < 0.05 involved SNPs located in lncRNA-75, and besides *GABPB1-AS1*, included only lincRNA AC022087.1 which lies downstream of *GABPB1-AS1* in reverse orientation. Two SNPs of the 18 that associate with *GBPB1-AS1* in GTEx also target *GABPB1-AS1* in the INQUISIT analysis: rs71124350 (*p* = 2.7 × 10^–7^, normalized effect size [NES] = −0.43) and rs28489579 (*p* = 1.6 × 10^–13^, NES = −0.52). The two SNPs are linked with *r*^2^ = 0.8996. All SNPs that show association with *GABPB1-AS1* expression are downstream of the gene starting from approximately 4.7kb. Rs71124350 lies 31kb and rs28489579 36kb from the gene.

### CPEB4 and TIAL1 Associate With SNPs Targeting Them in INQUISIT in eQTL Analysis of METABRIC Dataset

Of the 251 SNPs in this study, 20 had eQTL associations with the limit *p* < 0.05 in METABRIC (Supplementary Table S10). These SNPs were spread on three lncRNA regions and five T-UCRs (Supplementary Table S9). Even though the vast majority of the found associations were in cis, the specific lncRNAs or T-UCRs of the regions of interest were not associated with any of the SNPs. Altogether, the SNPs had 10322 associations ranging from 352 to 1151 associations per SNP. These associations contain 5858 genomic elements, including genes, pseudogenes, and expressed sequence tags. Each genetic element was associated with 1-17 SNPs. We focused on SNP and region associations with genes that were also INQUISIT-predicted target genes. There were two such genes, *CPEB4* and *TIAL1*.

Two SNPs, rs17695092 and rs1564823 in region uc.184, associated strongly in cis with *CPEB4*, *p* = 7.33 × 10^–61^ (after FDR correction 3.76 × 10^–55^) and *p* = 3.66 × 10^–64^ (after FDR correction 3.75 × 10^–58^) with beta coefficients of −0.317 and 0.324, respectively (Table 7). These SNPs have the lowest *p*-values of the METABRIC analysis and they are in strong linkage disequilibrium (*r*^2^ = 1.000). Both rs17695092 and rs1564823 as well as the T-UCR uc.184 are located within *CPEB4* gene: rs17695092 lies in the intron 2, while rs1564823 and uc.184 are situated in the 3′UTR of the gene.

Three SNPS, rs4752331, rs3009879, and rs12569630 in uc.313, associated in cis with *TIAL1* in METABRIC (Table 8). Only rs3009879 was predicted to target *TIAL1* by INQUISIT. The three SNPs are linked as *r*^2^ between rs3009879 and rs4752331 is 0.681, and between rs3009879 and rs12569630 *r*^2^ = 0.967. Rs3009879 is intronic, located in the *TIAL1*, while rs4752331 and rs12569630 are located 7.3 kb downstream and 6.1 kb upstream of the *TIAL1*, respectively. However, while the p-values range from 0.0013 to 0.0078, none survives FDR correction (all FDR corrected are *p*-values >0.9). The beta coefficient for rs4752331 and rs12569630 variants is 0.04, and for rs3009879 – 0.04.

### ncRNA eQTL Database Validates GABPB1-AS1 eQTL Association

To validate the *GABPB1-AS1* results from the GTEx eQTL analysis, we looked for other eQTL resources. Non-coding RNA eQTL database ncRNA eQTL was queried with the *GABPB1-AS1* GTEx eQTL results. The data base did not include the SNPs rs71124350 and rs28489579. However, SNPs in strong linkage disequilibrium with these SNPs of interest (*r*^2^ = 0.822 and 0.883, respectively) were found in the ncRNA eQTL database, and their results support the GTEx eQTL results: SNP rs35831049 linked to the SNP of interest rs71124350 (*r*^2^ = 0.822) was associated with *GABPB1-AS1* with effect *r* = −0.35 (rs71124350 normalized effect size [NES] = −0.43) and *p* = 9.29e-32, and SNP rs34565064 linked to rs28489579 (*r*^2^ = 0.883) was associated with *GABPB1-AS1* with effect *r* = −0.35 (rs28489579 NES = −0.52 and p = 5.45e-32).

## Discussion

In this study, we looked into the connection between lncRNAs and T-UCRs and breast cancer risk. The connection was investigated by identifying putative breast cancer risk SNPs in BCAC data located in or near lncRNAs and T-UCRs, assessing the SNPs’ functional effects using heuristic scoring method INQUISIT that predicts target genes for risk SNPs by combining genomic information from multiple sources, and performing eQTL analysis. These analysis methods are especially suitable for gaining insight into the role of SNPs located in the areas flanking the lncRNAs and T-UCRs and not directly affecting their sequence. All the SNPS found in this study to be associated with breast cancer were flanking SNPSs.

Of the 1303 breast cancer risk associated SNPs in 12 lncRNA-and 26 T-UCR loci in the study, 251 were in loci not previously reported by BCAC (7 lncRNA and 8 T-UCR), and for 60 of these in two lncRNA regions and 4 T-UCR, INQUISIT predicted a target gene. For three of these genes, also an eQTL association was found in METABRIC or GTEx eQTL analysis (Table 4). INQUISIT analysis predicted *GABPB1-AS1* as the target for two SNPs, rs71124350, and rs28489579, and the same SNP-gene association was seen in GTEx eQTL analysis of normal mammary tissue (Table 5). Results of a query to the ncRNA eQTL database support the eQTL association of *GABPB1-AS1*, and SNPS rs71124350 and rs28489579, although the database did not include these specific SNPs but others in strong linkage disequilibrium with them. In eQTL analysis of METABRIC breast cancer tissue data, *CPEB4* was found to be associated with SNP rs17695092, and the same SNP had *CPEB4* also as an INQUISIT target gene. Similarly, rs3008979 and *TIAL1* had METABRIC association and were a SNP-predicted target gene pair, although the p-values for *TIAL1* eQTL association did not survive FDR correction. In addition to these loci with functional data available, other candidate regions were identified containing SNPs with the defined p-value and highly significant FDR for breast cancer risk association (Supplementary Table S5).

The two *GABPB1-AS1* targeting SNPs, rs71124350 and rs28489579, are linked (*r*^2^ = 0.8996) and located near each other. According to a database of human enhancers, between the two SNPs lies a *GABPB1-AS1* enhancer site (GeneHancer ID GH15I050390). This site is not a direct enhancer of *GABPB1*, and concordantly rs71124350 and rs28489579 do not have an eQTL association with *GABPB1*. As the minor alleles of both rs71124350 and rs28489579 are also associated with a small decrease in breast cancer risk (Table 6), these findings suggest that the decrease in *GABPB1-AS1* expression associates with decreased breast cancer risk.

*GABPB1-AS1* is an lncRNA located in 15q21.2, partially overlapping *GABPB1* read from the opposite stand. There are reports of non-coding RNAs and the protein-coding genes they overlap displaying coordinated expression and function, which can be synergistic or antagonistic (39, 40). Commonly, the role of antisense RNAs is to bind the sense-oriented mRNA, and thus block its translation. There are no reports on how *GABPB1-AS1* affects the expression of *GABPB1*, but they share common promotor/enhancer loci according to GeneHancer: of the 18 promoter/enhancer regions associated with *GABPB1-AS1*, nine were also associated with *GABPB1*. *GABPB1* is a transcription factor and an activator of *BRCA1* expression (Atlas et al., 2000). If we assume the antisense – sense relationship between *GABPB1-AS1* and *GABPB1* to be an antagonistic one, it would suggest that *GABPB1-AS1* downregulates *GABPB1*, which in turn would lead to repression of *BRCA1*. This would be consistent with the results of this study: SNPs associated with reduced *GABPB1-AS1* are also associated with reduced breast cancer risk, and this effect could be the result of the increased *GABPB1* expression leading to increased *BRCA1* expression. However, *GABPB1-AS1* was selected for this study based on positive correlation between *GABPB1-AS1* and *BRCA1* expression. It is possible that the regulatory relationships are more complex than seen here, and the correlation between overall expression levels may not imply causation. Further research is required to clarify the functional interactions between these genes, as at this point, we can only speculate on the functional role of GABPB1-AS1in breast cancer predisposition.

For the other two discovered loci, the regions were included as T-UCR harboring loci but the discovered risk SNPs were associated in eQTL and INQUISIT analyses with protein coding genes: rs17695092 in uc.184 with *CPEB4*, and rs3009879 in uc.313 with *TIAL1*. Uc.184 and uc.313 are located in *CPEB4* and *TIAL1*, respectively. However, T-UCR expression is challenging to study, as they do not appear in expression databases. This is at least partly due to the difficulty in separating intragenic T-UCR expression from the expression of its host gene. Mestdagh et al. (2010) found uc.184 expression to be inseparable from *CPEB4* expression, while uc.313 expression was found to be independent of *TIAL1* expression. However, Mestdagh et al. looked at the expressions in neuroblastoma and the situation in breast tissue is unknown. Nevertheless, uc.187 and uc.313 are likely to play a substantial role in the correct function of their host genes, as such conservation is unlikely to remain intact by chance. Uc.184 and uc.313 are located in the 3′UTR and in an intron, respectively, and alterations in these regions often have a major regulatory effect on the function of a gene (Li and Yuan, 2017; Park et al., 2018). The fidelity of these regions may be essential to the correct function of the *CPEB4* and *TIAL1*.

The 3′UTR of the *CPEB4* contains 13 of the 17 SNPs that target *CPEB4* in INQUISIT prediction and one of the two SNPs with *CPEB4* expression association in METABRIC. T-UCR uc.184 is also located there. CPEB4 is a member of a CPEB family of proteins that bind RNA in a sequence-specific manner, contain two RNA recognition motifs, two zinc fingers and a regulatory N-terminal region (Hake and Richter, 1994; Fernandez-Miranda and Mendez, 2012). CPEBs regulate translation by controlling the polyadenylation of their target genes (Mendez and Richter, 2001; Richter, 2007). There are no previous reports of CPEB4 affecting breast cancer risk, but overexpression of *CPEB4* is reported in breast cancer, and the overall survival of patients with high expression of *CPEB4* is shorter (Sun et al., 2015; Lu et al., 2017). Ectopic *CPEB4* expression has been suggested to promote EMT, migration and invasion of breast cancer cells, while silencing the expression of *CPEB4* reduces these events (Lu et al., 2017). Our results imply that CPEB4 may also play a role in the breast cancer development as the intronic SNP rs17695092 associates with both reduced *CPEB4* expression, and reduced breast cancer risk (Table 7). It is to be noted that the METABRIC dataset consists of breast cancer samples, and the effect is not seen in the eQTL analysis in the GTEx dataset of normal mammary tissue samples. The difference could be due to difference in statistical power, as the METABRIC dataset includes over 1300 breast cancer samples, whereas the GTEx dataset is 251 normal breast cancer tissues. It is notable that the cell line used in the CHiA-PET analysis from which the INQUISIT results for rs17695092 were gathered was MCF7, which is a breast cancer cell line. This requires further research, as does the role of the uc.184 in the 3′UTR of the *CPEB4*.

Uc.313 is located in the intron 5 or 6 of the *TIAL1*, depending on the transcript (and in a single transcript, NM_001323964.1, out of the eleven UCSC annotations of the RefSeq RNAs, it partially overlaps exon six). Of the twelve SNPs that target *TIAL1* in INQUISIT prediction, or as METABRIC association, the majority are located downstream of the gene, three are in the *TIAL1*, all intronic, and one is located upstream of the gene (Table 8). The SNP with *TIAL1* as both INQUISIT target and METABRIC association, rs3009879, is one of the three intronic variants. Rs3009879 does not appear to overlap any regulatory sequence elements (assessed by using Ensembl genome browser 92 and GeneHancer in GeneCards), but as it does target *TIAL1* in INQUISIT, a connection discovered by the CHiA-PET method, it suggests involvement in a chromatin interaction. It is worth noting that in METABRIC eQTL analysis, the significance of rs3009879 association with*TIAL1* expression was *p* = 0.0013, but it did not survive FDR correction. Thus, it is also possible that the eQTL association of this variant with*TIAL1* is an artefact.

*TIAL1* (also known as *TIAR*), is a ubiquitously expressed RNA binding protein that contains three N-terminal RNA recognition motifs and a C-terminal glutamine-rich prion-like domain, which is found to aggregate during the formation of cytoplasmic stress granules (Dember et al., 1996; Gilks et al., 2004; Kim et al., 2013). *TIAL1* is a negative regulator of *BRCA1*: it is shown to block translation, and at least in chronic myeloid leukemia cells, reduce the protein expression of *BRCA1* which leads to aneuploidy, spindle toxin resistance, and genomic instability (Deutsch et al., 2003; Wolanin et al., 2010; Podszywalow-Bartnicka et al., 2014). If *TIAL1* has the same effect on *BRCA1* protein expression in breast cancer, it is plausible that SNPs that increase *TIAL1* expression also increase breast cancer risk, as is the case with rs3009879 (Supplementary Table S9).

Previously, SNPs with genome-wide significant associations (*p* < 5 × 10^–8^) with breast cancer risk have been reported in several genomic regions containing lncRNAs (Michailidou et al., 2017; Milne et al., 2017). In this study, we aimed to identify additional candidate loci for further studies. We report here putative breast cancer risk SNPs predicted to functionally target *GABPB1-AS1* lncRNA, and associating with its expression, as well as SNPs in two genes, *CPEB4* and *TIAL1*, hosting ultraconserved regions, uc.184 and uc.313, respectively. Further research is needed to validate these findings and candidate genes, and elucidate the functional mechanisms involved. In addition, other regions containing SNPs with the defined *p*-value and highly significant FDR for breast cancer risk association, but currently lacking the functional data, may warrant further studies.

## Data Availability Statement

The analyses were based on summary results of the Breast Cancer Association Consortium (BCAC), available online at: http://bcac.ccge.medschl.cam.ac.uk/.

## Ethics Statement

All participating BCAC studies were approved by their appropriate institutional ethics review boards for the initial BCAC study. This study uses only publicly available BCAC-summary data, no individual data.

## Author Contributions

SK and HN designed the study. MS and SK carried out the data and eQTL analyses, wrote the main manuscript text and prepared the figures and the tables. CB provided clinical expertise and critically reviewed the manuscript. JB and GC-T provided the INQUISIT analysis. All authors contributed to and approved the final manuscript.

## Conflict of Interest

The authors declare that the research was conducted in the absence of any commercial or financial relationships that could be construed as a potential conflict of interest.

## Footnotes

1 http://bcac.ccge.medschl.cam.ac.uk/

2 https://www.gtexportal.org/home/

3 https://ega-archive.org/

4 http://ibi.hzau.edu.cn/ncRNA-eQTL/

5 http://bcac.ccge.medschl.cam.ac.uk/

6 http://www.R-project.org/

7 www.genecards.org

8 https://genome.ucsc.edu/

9 http://www.ensembl.org
